# Unplanned hospital transfers from nursing homes: who is involved in the transfer decision? Results from the HOMERN study

**DOI:** 10.1007/s40520-020-01751-5

**Published:** 2020-11-30

**Authors:** Alexandra Pulst, Alexander Maximilian Fassmer, Guido Schmiemann

**Affiliations:** 1 Department for Health Services Research, Institute of Public Health and Nursing Research, University of Bremen, Grazer Straße 4, 28359 Bremen, Germany; 2 Health Sciences Bremen, University of Bremen, 28359 Bremen, Germany; 3 Department of Health Services Research, School VI, Medicine and Health Sciences, Carl von Ossietzky University of Oldenburg, Ammerländer Heerstraße 114-118, 26129 Oldenburg, Germany; 4 Institute for General Practice, Hannover Medical School, 30625 Hannover, Germany

**Keywords:** Nursing home residents, Hospitalization, Hospital admission, Patient transfer, Emergency department, Decision making

## Abstract

**Background:**

Emergency department visits and hospital admissions are common among nursing home residents (NHRs) and seem to be higher in Germany than in other countries. Yet, research on characteristics of transfers and involved persons in the transfer decision is scarce.

**Aims:**

The aim of this study was to analyze the characteristics of hospital transfers from nursing homes (NHs) focused on contacts to physicians, family members and legal guardians prior to a transfer.

**Methods:**

We conducted a multi-center study in 14 NHs in the regions Bremen and Lower Saxony (Northwestern Germany) between March 2018 and July 2019. Hospital transfers were documented for 12 months by nursing staff using a standardized questionnaire. Data were derived from care records and perspectives of nursing staff and were analyzed descriptively.

**Results:**

Among 802 included NHRs, *n* = 535 unplanned hospital transfers occurred of which 63.1% resulted in an admission. Main reasons were deterioration of health status (e.g. fever, infections, dyspnea and exsiccosis) (35.1%) and falls/accidents/injuries (33.5%). Within 48 h prior to transfer, contact to at least one general practitioner (GP)/specialist/out-of-hour-care physician was 46.2% and varied between the NHs (range: 32.3–83.3%). GPs were involved in only 34.8% of transfer decisions. Relatives and legal guardians were more often informed about transfer (62.3% and 66.8%) than involved in the decision (21.8% and 15.1%).

**Discussion:**

Contacts to physicians and involvement of the GP were low prior to unplanned transfers. The ranges between the NHs may be explained by organizational differences.

**Conclusion:**

Improvements in communication between nursing staff, physicians and others are required to reduce potentially avoidable transfers.

**Electronic supplementary material:**

The online version of this article (10.1007/s40520-020-01751-5) contains supplementary material, which is available to authorized users.

## Introduction

Nursing home residents (NHRs) are characterized by multimorbidity and frailty [1]. Compared to community-dwelling older people, transfers to emergency department (ED) and hospital admissions are more frequent among NHRs [2–6]. The prevalence of transfers from nursing homes (NHs) ranges in international studies between 6.8% and 45.7% depending on the study design and different time periods of follow-up [5] and seem to be more common in Germany than in other Western countries [7–9]. Transfers can lead to adverse consequences like nosocomial infections, delirium or functional decline and high costs due to increased ambulance use and hospital treatment [2, 10].

In Germany, medical care of NHRs is usually provided by general practitioners (GPs) [11].If specialized care is required, treatment in NHs can be provided by specialists. Elderly care physicians as in the Netherlands, for example [12] are an exception. In the case of health deteriorations outside of GPs’ working hours, nursing staff can contact out-of-hour health care services (OOHC) in which every practicing physician is legally obliged to participate [13]. In Germany, the OOHC can be called by the nationwide phone number “116 117”.In life-threatening cases, the Emergency Medical Service (EMS) is responsible and can be contacted to send out an ambulance (“112”). Even though there are several possibilities to manage health changes of NHR in outpatient care, ambulance calls increased in NHs over the last years [14, 15]. In this context, several studies indicate that some hospitals transfers from NHs might be avoidable [16–18].

The process from the onset of health changes to the transfer decision is complex and can be influenced by different factors. Studies analyzing transfers of NHRs often follow a retrospective design [19–21]. In the literature individual factors (e.g. clinical conditions [2], degree of frailty [22], availability of advance directives (ADs) [23]), structural factors (e.g. staffing capacity [24, 25], access to equipment in NH [23], availability of physicians [10]) and communication deficits between nursing staff and physicians [23] are described as reasons for hospital transfers from NHs. Additionally, significant others such as family members and legal guardians can influence the transfer decision [26, 27].

So far, it is unclear how often physicians, family members and legal guardians are involved before an ambulance is called. This is especially relevant in unplanned transfers which can be initiated in Germany without prior medical order of a physician. As a result of this lacking reassurance, many ambulance calls can lead to hospital transports and admissions. Therefore unplanned transfers may be a major contributor for potentially avoidable hospital transfers from NHs. In Germany, evidence on characteristics of unplanned hospital transfers from NHs and involvement of others is scarce. Therefore, this study intends to close this gap in research. The aim was to explore the characteristics of unplanned hospital transfers from NHs and to analyze prior contacts to different health care providers and involvement of others in the transfer decision.

## Methods

### Study design

We used data from the project ‘HOspitalisations and eMERrgency department visits of Nursing home residents’ (HOMERN). In this project, we conducted a multi-center observational study in NHs providing long-term care in Northwestern Germany. The convenience sample consisted of NHs in the region to which we had personal contacts and/or who had participated in former research projects and further facilities. They differed in size (numbers of beds), ownership (non-profit and private for-profit) and location (rural and urban) to achieve heterogeneity. All NHs were located in the federal state Bremen and surrounding Lower Saxony (Northwestern Germany). Because we focused on long-term care NHs, we excluded short-term care units in this study. We further excluded NHs that offered specialized care only (e.g. for residents with dementia) to reduce the risk of selection effects and overestimation of dementia in the study population. Participating NHs were asked to include either all residents of the whole NH or alternatively all residents living in selected care units. There were no exclusion criteria for the residents. In total, *n* = 49 NHs were contacted for recruitment of whom *n* = 14 NHs agreed to participate.

During a 12-month period, data on all (un)planned hospital transfers (including ED visits and hospital admissions) were collected in the 14 NHs from March 2018 to July 2019. Nursing staff (mainly nursing managers) in the NHs were responsible for data collection and trained to answer the questionnaire. Data included clinical information from medical records and the perspectives of nursing staff only. Informed consent was obtained from all participating NHs before the study started. Residents did not participate actively in the study. Ethical approval for this study was given by the ethics committee of the Medical Association of Bremen (RA/RE-613, 16 February 2018). This article followed the STROBE guidelines for reporting observational studies [28].

### Data collection and analysis

Based on existing literature [10, 16, 18, 24, 26, 29] a standardized questionnaire was developed to collect characteristics of hospital transfers and persons involved in transfer decision. The questionnaire was pilot-tested with nursing managers in three NHs which also participated in the study later. Based on their comments the questionnaire was revised. Each NH assigned a contact person (mainly nursing managers) who was responsible for data collection and trained in the handling of the questionnaire prior to the study.

For each transfer nursing staff was asked to document: characteristics of the NHR (age, sex, marital status, duration of NH residence, frequency of transfers in the last 12 months), impairment in activities of daily living (Barthel-Index), and availability/content of ADs. As dementia is often underreported in medical records [30, 31], diagnosis and severity of dementia were assessed by nursing staff.

Characteristics of the transfer included date, time-slot, death during transfer/in hospital, the result of the transfer (ED visit/hospital admission), kind/duration of symptoms and date of discharge. Further, we assessed contacts to GPs, specialists, OOHC, EMS and emergency physicians in the last 48 h prior to transfer (‘yes—via telephone’ or ‘yes—via NH visit’ or ‘no contact’). Involvement of the resident, family members and legal guardians in transfer decision was enquired using a 4-point Likert scale (‘was involved in transfer decision’, ‘was informed about transfer’, ‘not contacted’ or ‘involvement unknown’). Family members and legal guardians could also be assessed as ‘not existent’. The involvement of the GP in transfer decision was assessed by four categories: ‘involved via NH visit’, ‘involved via telephone’, ‘not involved because not available’, ‘not involved because not contacted’. Data on size and staffing of NHs were not collected, as an individual analysis was not planned due to data confidentiality. Further open questions were used for assessment of symptoms (which were not covered by the given categories) and the specialization of an involved specialist. The staff was asked to fill out the questionnaire immediately after each transfer. For each documentation, a financial compensation of 10 Euro was paid. There was regular personal and telephone contact between researchers and NHs to ensure that all transfers were documented and that questionnaires were picked up regularly.

The data were descriptively analyzed using IBM SPSS Statistics 25 and SAS 9.4 (SAS Institute, Cary, USA). Because not all respondents answered every question in the questionnaire, the analyses of each question were based on subjects without missing values. Below, we report on unplanned hospital transfers only. An analysis considering also planned transfers is already published elsewhere [32].

## Results

In total, 14 NHs with *n* = 802 residents were included. Mean number of included residents per NH was 57 (range: 26–114). During 12 months of follow-up, *n* = 626 hospital transfers occurred. N = 3 transfers were excluded from data analysis because these residents died during transfer and no further information was provided. 85.9% of transfers were unplanned (*n* = 535). Median rate for unplanned transfers was 0.67 per resident and year.

### Characteristics of transferred NHRs

Transferred residents were mainly female (70.2%) and widowed (63.7%). Mean age was 83.8 years (range: 49–103 years). Mean duration of NH residence was 2.4 years (see Table 1). More than half of the residents had one to three hospital transfers (51.8%) in the last 12 months before study inclusion, 11.3% were transferred more than three times and 36.9% had not been hospitalized in the year before.

**Table 1:** Characteristics of unplanned transferred nursing home residents (*n* = 535)

		*N* (%)^a^
Sex	Total	(*n* = 534)^a^
	Male	159 (29.8)
	Female	375 (70.2)
Age (years)	Total Mean (SD) [range]	(*n* = 531)^a^ 83.8 (9.3) [49–103]
	0–69	42 (7.9)
	70–79	95 (17.9)
	80–89	252 (47.5)
	90 +	142 (26.7)
Marital status	Total	(*n* = 523)^a^
	Widowed	333 (63.7)
	Married/living in partnership	95 (18.2)
	Single	47 (9.0)
	Divorced/separate	48 (9.2)
Length of NH residence	Total Mean (SD) [range]	(*n* = 518)^a^ 2.4 (2.7) [0–24]
	< 1 year	200 (38.8)
	1 to < 2 years	95 (18.4)
	2 to < 3 years	67 (12.6)
	≥ 3 year	156 (30.2)
Dementia	Total	(*n* = 532)^a^
	No	259 (48.7)
	Yes	273 (51.3)
	Mild	44 (16.6)
	Moderate	124 (46.8)
	Severe	97 (36.6)
Barthel-Index (ADL)^b^	Total Mean (SD) [range]	(*n* = 517)^a^ 43.3 (24.9) [0–100]
	Slight/no dependency (score: 80–100)	43 (8.3)
	Mild dependency (score: 60–75)	123 (23.8)
	Moderate dependency (score: 40–55)	141 (27.3)
	Severe dependency (score: 20–35)	105 (20.3)
	Total dependency (score: 0–15)	105 (20.3)
Residents’ wish for end-of-life care	Total	(*n* = 528)^a^
	Unknown	282 (53.1)
	Advance directive available	249 (46.9)

*NH* nursing home, *ADL* Activities of Daily Living

^a^Number differs due to missing values

^b^Based on ICD 10-GM Version 2016, see: https://www.dimdi.de/static/de/klassifikationen/icd/icd-10-gm/kode-suche/htmlgm2016/block-u50-u52.htm

### Characteristics of unplanned transfers

**Fig. 1:**
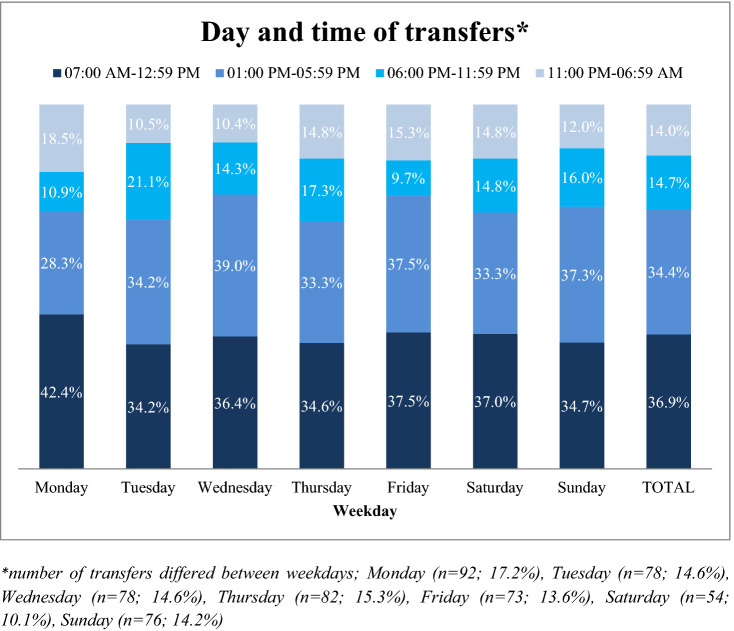
Weekday and time of transfers (*n* = 533). *Number of transfers differed between weekdays; Monday (*n* = 92; 17.2%), Tuesday (*n* = 78; 14.6%), Wednesday (*n* = 78; 14.6%), Thursday (*n* = 82; 15.3%), Friday (*n* = 73; 13.6%), Saturday (*n* = 54; 10.1%), Sunday (*n* = 76; 14.2%)

Transfers were nearly equally spread over the weekdays (ranges between 10.1% and 17.3%) with a maximum on Mondays (17.3%; *n* = 92). 24.4% of transfers were initiated on weekends. NHRs were mainly transferred during the morning (36.9%) or early afternoon (34.4%) (Fig. 1).

Main reasons for transfer were a deterioration of health status including fever, infections, dyspnea and exsiccosis (35.1%) and falls/accidents/injuries (33.5%) (see Supplementary file 1). Most symptoms occurred in the 4 h prior to transfer (60.4%) and in 14.7% the symptoms lasted more than 24 h. In the majority of cases, transfers lead to admission (63.1%), while 36.9% of treatments were carried out in ED. Hospital admission were mainly required because of deterioration of health status (48.5%), ED visits were mostly required because of falls/accidents/injuries (51.8%).

### Contacts to health care provider 48 h prior to transfer

48 h prior to the transfer, nursing staff contacted the GP in 38.7% (via telephone only: 17.1%; via NH visit: 21.6%). The extent to which the GPs were consulted prior to transport differed between the underlying symptoms: GPs were mostly involved in case of a deteriorated health status (52.1%), pain not induced by fall (51.5%) and others symptoms like gastrointestinal symptoms or bleedings (50.9%).

In 8.4% nursing staff contacted a specialist (only via telephone: 4.1%; via NH visit: 4.3%) and in 7.4% OOHC (only via telephone: 5.1%; via NH visit: 2.3%). Contact rate to at least one physician (GP, specialist, OOHC) was 46.2% and varied between the NHs (range: 32.3–83.3%). In every fifth case (17.0%), nursing staff called the EMS/emergency physician directly (see Table 2). In 44.5% no health care provider was contacted (range between NHs: 5.9–63.0%). For further information see Table 2 (only transfers with prior contacts are displayed).

**Table 2:** Contacts to physicians and other health care providers within *48 h prior to transfer*– by resident and transfer characteristics^a^

	GP		Specialist		OOHC		EMS/EP	
	*N*		*N*		*N*		*N*	
Contacts prior to all transfers	206	(38.7%)	45	(8.4%)	39	(7.3%)	91	(17.0%)
By resident’s sex	*N* = 206		*N* = 45		*N* = 39		*N* = 91	
Male	56	(35.4%)	21	(13.3%)	11	(7.0%)	29	(18.2%)
Female	150	(40.2%)	24	(6.4%)	28	(7.5%)	62	(16.5%)
By resident’s age	*N* = 205^a^		*N* = 45		*N* = 39		*N* = 91	
0–69	15	(35.7%)	4	(9.5%)	4	(9.5%)	10	(23.8%)
70–79	33	(35.1%)	12	(12.6%)	4	(4.3%)	16	(16.8%)
80–89	107	(42.6%)	21	(8.4%)	22	(8.8%)	39	(15.5%)
90 +	50	(35.5%)	8	(5.7%)	9	(6.4%)	26	(18.3%)
By result of transfer	*N* = 202^a^		*N* = 45		*N* = 39		*N* = 91	
ED visit	62	(32.0%)	22	(11.3%)	8	(4.1%)	33	(16.9%)
Hospital admission	140	(42.2%)	23	(6.9%)	31	(9.4%)	58	(17.4%)
By weekday of transfer	*N* = 206		*N* = 45		*N* = 39		*N* = 89^a^	
Monday	37	(40.7%)	7	(7.6%)	8	(8.8%)	14	(15.2%)
Tuesday	30	(38.5%)	5	(6.4%)	3	(3.9%)	15	(19.2%)
Wednesday	33	(42.3%)	10	(12.8%)	6	(7.8%)	15	(19.2%)
Thursday	46	(56.1%)	6	(7.3%)	0	(0.0%)	10	(12.2%)
Friday	31	(43.7%)	6	(8.5%)	2	(2.8%)	8	(11.0%)
Saturday	14	(25.9%)	5	(9.3%)	10	(18.5%)	10	(18.5%)
Sunday	15	(19.7%)	6	(7.9%)	10	(13.2%)	17	(22.4%)
By time of transfer	*N* = 204^a^		*N* = 45		*N* = 39		*N* = 91	
07:00am–12:59 pm	79	(40.7%)	6	(3.1%)	14	(7.2%)	31	(15.9%)
01:00 pm–05:59 pm	83	(45.9%)	27	(14.9%)	12	(6.7%)	31	(17.0%)
06:00 pm–10:59 pm	25	(32.1%)	6	(7.7%)	5	(6.4%)	13	(16.7%)
11:00 pm–06:59am	17	(23.3%	6	(8.2%)	8	(11.0%)	16	(21.6%)
By reasons (symptoms)	*N* = 206		*N* = 45		*N* = 39		*N* = 91	
Deterioration of health status (e.g. fever, infection, dyspnea, exsiccosis)	98	(52.1%)	9	(4.8%)	19	(10.1%)	31	(16.5%)
Fall/accident/injury	41	(23.0%)	14	(7.9%)	4	(2.3%)	28	(15.6%)
Psychiatric/neurologic disorders (e.g. challenging behavior, stroke)	14	(36.8%)	5	(13.2%)	1	(2.6%)	8	(21.1%)
Complications with catheter/tube (e.g. blood in urine)	7	(18.4%)	10	(26.3%)	3	(7.9%)	10	(26.3%)
Pain, not fall-induced	17	(51.5%)	1	(3.0%)	4	(12.1%)	5	(15.2%)
Others (e.g. gastrointestinal symptoms, bleedings)	29	(50.9%)	6	(10.3%)	8	(14.3%)	9	(15.3%)
By duration of symptoms	*N* = 205^a^		*N* = 45		*N* = 38^a^		*N* = 88^a^	
Less than 4 h	78	(24.5%)	23	(7.2%)	17	(5.4%)	52	(16.3%)
Between 4 and 12 h	41	(53.3%)	6	(7.8%)	8	(10.4%)	13	(16.9%)
Between 12 and 24 h	31	(57.4%)	5	(9.3%)	7	(13.0%)	13	(24.1%)
Between 25 and 72 h	19	(76.0%)	2	(8.0%)	4	(16.0%)	3	(12.0%)
Longer than 72 h	36	(70.6%)	9	(17.3%)	2	(3.9%)	7	(13.2%)
By residents’ wish for end-of-life care	*N* = 205^a^		*N* = 45		*N* = 38^a^		*N* = 90^a^	
Unknown	108	(38.4%)	22	(7.8%)	23	(8.2%)	49	(17.4%)
Advance directive available	97	(39.3%)	23	(9.3%)	15	(6.1%)	41	(16.5%)

*EMS* Emergency Medical Services, *EP* emergency physician, *GP* general practitioner

^a^Only transfers with prior contacts are displayed, multiple contacts possible; numbers varied due to missing values

For example: GPs were contacted in 35.4% of transfers when residents were male

### Involvement in the transfer decision

In two-thirds of the cases (65.2%), the GP was not involved in the transfer decision—either because nursing staff did not try to contact him/her (38.3%) or GP was not available (26.9%). Between the NHs, this non-involvement varied (range: 5.9–73.9% because of no contact and 0.0–47.1% because of lacking availability). Related to the symptom duration, GPs were more often involved when symptoms lasted 24 h or longer (71.8%) in contrast to symptoms lasting less than 4 h (17.7%). NHRs themselves were either involved in the decision (45.8%) or only informed (50.0%). In 93.7% (*n* = 472) NHRs had family members. These were involved in 21.8% (range between NHs: 7.1–55.5%) or only informed in 62.3% (range between NHs: 38.9.3–92.8%). If legal guardians were available (*n* = 238), they were informed only in 66.8% (directly involved in 15.1%). For details see Table 3 (only transfers with prior involvement are displayed).

**Table 3:** Involvement of GPs, residents, family members and legal guardians in transfer decision—by resident and transfer characteristics^a^

	GP^b^		Resident^c^		Family member^c^		Legal guardian^c^	
	*N*		*N*		*N*		*N*	
Involvement in all transfer decisions	185	(34.8%)	498	(95.8%)	397	(78.8%)	195	(42.4%)
Involvement by								
Resident’s sex	*N* = 185		*N* = 498		*N* = 396^a^		*N* = 194^a^	
Male	53	(33.5%)	149	(94.9%)	114	(75.5%)	59	(43.1%)
Female	132	(35.3%)	349	(96.1%)	282	(80.1%)	135	(41.9%)
Resident’s age	*N* = 185		*N* = 494^a^		*N* = 393^a^		*N* = 193^a^	
0–69	13	(31.7%)	38	(90.5%)	25	(64.1%)	19	(50.0%
70–79	26	(27.7%)	84	(96.6%)	66	(75.0%)	35	(45.4%)
80–89	93	(37.1%)	238	(96.4%)	191	(81.6%)	99	(46.0%)
90 +	53	(37.3%)	134	(95.7%)	111	(79.9%)	40	(31.5%)
Result of transfer	*N* = 181^a^		*N* = 493^a^		*N* = 392^a^		*N* = 192^a^	
ED visit	46	(23.7%)	184	(96.3%)	127	(71.7%)	57	(35.2%)
Hospital admission	135	(40.7%)	309	(95.7%)	265	(82.3%)	135	(45.9%)
Weekday of transfer	*N* = 185		*N* = 498		*N* = 396^a^		*N* = 194^a^	
Monday	31	(33.7%)	86	(94.5%)	63	(73.3%)	29	(36.7%)
Tuesday	33	(42.3%)	70	(90.9%)	58	(78.4%)	29	(43.9%)
Wednesday	32	(41.0%)	73	(97.3%)	63	(85.1%)	26	(37.7%)
Thursday	39	(47.6%)	80	(98.8%)	63	(82.9%)	36	(50.7%)
Friday	31	(43.7%)	68	(98.6%)	49	(73.1%)	24	(38.7%)
Saturday	8	(14.8%)	50	(94.3%)	38	(74.5%)	17	(36.2%)
Sunday	11	(14.5%)	71	(97.3%)	62	(83.8%)	33	(51.6%)
Time of transfer	*N* = 182^a^		*N* = 492^a^		*N* = 392^a^		*N* = 192^a^	
07:00am–12:59 pm	84	(43.5%)	182	(96.3%)	149	(78.8%)	84	(47.2%)
01:00 pm–05:59 pm	80	(44.0%)	170	(95.5%)	142	(83.5%)	66	(43.1%)
06:00 pm–10:59 pm	16	(20.5%)	74	(97.4%)	60	(82.2%)	27	(41.5%)
11:00 pm–06:59am	2	(2.7%)	66	(93.0%)	41	(61.2%)	15	(25.4%)
Reasons (symptoms)	*N* = 185		*N* = 498		*N* = 397^a^		*N* = 195	
Deterioration of health status (e.g. fever, infection, dyspnea, exsiccosis)	98	(52.4%)	174	(94.6%)	139	(77.2%)	76	(46.6%)
Fall/accident/injury	29	(16.3%)	171	(97.7%)	131	(79.9%)	59	(38.1%)
Psychiatric/neurologic disorders (e.g. challenging behavior, stroke)	13	(35.1%)	33	(94.3%)	32	(86.5%)	20	(57.1%)
Complications with catheter/tube (e.g. blood in urine)	6	(15.8%)	36	(97.3%)	23	(63.9%)	7	(22.6%)
Pain, not fall-induced	15	(45.5%)	32	(97.0%)	26	(83.9%)	12	(44.4)
Others (e.g. gastrointestinal symptoms, bleedings)	24	(40.7%)	52	(92.9%)	46	(82.1%)	21	(42.9%)
Duration of symptoms	*N* = 184^a^		*N* = 492^a^		*N* = 393^a^		*N* = 192^a^	
Less than 4 h	56	(17.7%)	296	(96.1%)	230	(78.2%)	103	(38.4%)
Between 4 and 12 h	42	(54.6%)	71	(93.4%)	63	(82.9%)	36	(50.0%)
Between 12 and 24 h	30	(55.6%)	52	(96.3%)	42	(79.2%)	21	(44.7%)
Between 25 and 72 h	19	(76.0%)	22	(91.7%)	20	(83.3%)	9	(42.9%)
Longer than 72 h	37	(69.8%)	51	(98.1%)	38	(74.5%)	23	(48.9%)
Residents’ wishes for end-of-life care	*N* = 184^a^		*N* = 496^a^		*N* = 394^a^		*N* = 194^a^	
Unknown	100	(35.7%)	266	(95.7%)	194	(72.7%)	112	(44.3%)
Advance directive available	84	(33.9%)	230	(95.8%)	200	(85.8%)	82	(40.0%)

*EMS* Emergency Medical Services, *EP* emergency physician, *GP* general practitioner

^a^Only transfers with involvement of the GP, residents, family members and legal guardians are displayed; multiple answer possible; numbers varied due to missing values

For example: GPs were involved in 33.5% of transfer decision when residents were male

^b^GP involved via telephone or NH visit

^c^Involved in decision or informed about transfer

Focusing on transfers which were carried out during working hours when GPs and specialists are potentially available for consultation (Monday, Tuesday, Thursday, Friday 08:00am–05:59 pm and Wednesday 08:00am–12:59 pm), we could identify *n* = 254 transfers (47.5%). Nevertheless, in only 53.5% of those transfers the GPs were involved in the final transfer decision (via telephone: 31.3%, via NH visit: 22.2%). In 30.2% nursing staff did not make any attempt to contact the GP and in 16.3% the GP was not available. These rates of non-involvement varied also among the NHs and ranged between 0.0% and 54.5% (because of no contact) and 0.0–37.5% (because of lacking availability).

## Discussion

In our study, we assessed the characteristics of unplanned hospital transfers of NHRs and circumstances prior to a transfer. Deterioration of health status and falls/accidents/injuries were identified as the main reasons leading to a transfer. The majority of transfers happened without the involvement of the GP and the relevance of other physicians (specialists or OOHC) was low. We found that NHRs themselves were involved in the transfer decision in 45.8% of cases. Family members and legal guardians were more often informed about the transfer rather than involved in the decision.

### Physician contacts and involvement of the GP

Deteriorations of health status and falls were also reported in other studies as reasons for hospital transfers among NHRs [4, 16, 33]. These symptoms can often be managed in ambulatory care. However, the management by physicians appears to be low in our study. In nearly half of the cases, there was no prior contact to any health care provider in the 48 h prior to an ambulance call and residents were directly transferred to hospital. This is in line with findings reported by Briggs et al. [34]. In this context, low contacts to specialists and the OOHC seem to be not surprising due to the fact that NH visits by specialists are—depending on their specialization—insufficient in Germany [35, 36] and the financial compensation for participating in the OOHC is criticized as too low by physicians [13]. Both facts may contribute to the low presence/relevance of these physicians in the management of NHRs’ health changes.

Even without a formal gatekeeper system in Germany, the GP is primarily responsible for care decisions of NHRs. However, in our study GPs were contacted in the 48 h prior to a transfer in only 38.7% of cases. Later during transfer decision, the GP was involved in only every third case. This non-involvement cannot be traced back on life-threatening conditions which require quick reactions. In our study, mainly falls were reasons for transfers which do not always require hospital care. Other studies discuss in this context the insufficient availability of physicians [16, 32]. We found that even during GP working hours—when availability can be assumed—nursing staff did not make any attempt to contact the GP for a final transfer decision in 30.2% of cases. Similar observations of low pre-transfer contacts to the GP were reported in several other studies [29, 34, 37–39]. Because the EMS in Germany is not allowed to make a medical diagnosis, they are often trapped in a situation of uncertainty. Being send by a dispatcher, without official authority to make a diagnosis they are obliged to transport every patient to hospital [40]. We also found high differences between the NHs. These marked differences indicate the impact of organizational factors contributing to unplanned transfers. Günther et al. [41] showed in this context that the number of involved physicians, intervals of routine visits or presence of advance care planning (ACP) might have an impact on physicians’ involvement and transfers of NHRs. However, we have no detailed information on NH characteristics (staffing, for example). Other contributing factors might be the workload and internal instructions in each facility. When calling an ambulance, the NHR will be transferred to hospital without the need of further waiting, discussing and organizing a contact with a physician. Nursing staff might therefore tend to get rid of some residents and can consequently also avoid additional care [16, 42]. This problem might be more apparent in NHs with high workload and nursing staff with limited competence than in NHs which have the capacities to manage symptoms on-site. Additionally, the fear of legal consequences can be represented differently in the NH which is often a main trigger for nursing staff to call the ambulance [32, 43, 44]. Transfers initiated based on these reasons might be avoidable [20, 45, 46].

### Involvement of NHRs, family members and legal guardians

In view of nursing staff, NHRs’ wishes and quality of life are rated as highly influential for transfer decision [47, 48]. In our study, NHRs themselves were more informed about the upcoming transfer than involved in the decision. On the one hand, the high prevalence of dementia (51.3%) can have an influence when the need for a transfer and residents’ wishes cannot be communicated adequately. On the other hand, ADs with documented residents’ wishes for end-of-life care were available in just 46.9%. In more than half of the cases, nursing staff was therefore faced with the challenge to act in the NHRs’ best interest without knowledge about their preferences. This problem was also reported in other studies [16, 49]. As a consequence, nursing staff could mainly rely on the view of family members and legal guardians which were considered in several studies as having high influence on transfer decisions [27, 47, 50, 51]. However, in our study family members were more informed about the transfer than actually involved in the decision (62.3% vs. 21.8%). A main reason could be the short time period between the onset of symptoms and initiation of the transfer, which mainly lasted less than 4 h (60.4%). More importantly, we do not know if this non-involvement may be desired by family members. We assume that structured ACP discussions in NHs are fundamentally important to communicate and document preferences of residents and family members together with nursing staff and physicians. This implies more than the existence of ADs because they alone cannot guarantee a prevention of a hospital transfer [52, 53]. But so far, the concept of ACP is not established in Germany in contrast to other countries, such as the USA or Canada [54].

### Implications for practice

Main findings are a low degree of GP involvement in transfer decisions and marked differences between nursing homes regarding the involvement. Structural differences might explain these findings and can be part of the solution at the same time. In Germany, NHRs are cared for by an average of 8.6 different GPs [35]. A reduced number of responsible GPs might improve the collaboration and coordination of care leading to a reduction of transfers from NHs [27]. In the Netherlands for example, ‘elderly care physicians’ with a 3-year training program are employed in NHs to care for all residents on-site [12, 55, 56]. They have the potential to monitor health changes more closely which can prevent deteriorations of health status. In Germany, there is one model project which realized this concept of a NH physician and showed a reduction of hospital transfers and health care costs [57, 58]. However, it is still not part of regular health care. In other countries, nurse practitioners or specialist nurses, as established in the USA for example [12, 35, 55, 56] perform in the first-line assessment of residents which is correlated with fewer hospital transfers [59–62]. In Germany, these professional groups are not established due to the legal right to choose the GP freely and nursing staffs’ limited rights in medical care. Because nursing staff often fear legal consequences [32, 43], we assume that legal relaxations of personal liability could encourage nursing staff to more responsibility leading to a reduction of transfers. In general, there is a need for more personal in NHs to decrease workload, as criticized in other studies as a reasonfor hospital transfers [32].

## Strengths and limitations

With our study we provide current data on hospital transfers from NHs in Germany. However, some limitations have to be considered. Due to data anonymization, we can only provide information based on transfers (and not on residents). Further, we have no information on not-transported residents. Analysis based on resident-level (for example frequency of multiple transfers or the relationship between age/frailty and transfers) was not possible—but also not the aim of the study. A high proportion of cases were documented in three NHs because all residents of these facilities were included. This may increase the risk of selection bias. Even though most information was based on existing medical records, a recall-bias cannot be excluded. Additionally, there might the risk of an underreporting when nursing staff have forgotten to document a transfer. We therefore reminded nursing staff regularly to document each transfer immediately after each event. Despite recruiting NHs with different locations, sizes and ownerships, a selection of the sample is still possible due to voluntary participation and payment of financial compensation. The generalizability of our data can be therefore limited—also in comparison to other regions of Germany or health care systems in other countries. Nevertheless, this is the first study providing insights into contacts to physicians and involvement of relevant others prior to a transfer from NHs.

## Conclusions

Our study provides recent data on hospital transfers of NHRs in Germany. Specialists and OOHC physicians are rarely contacted prior to an ambulance call and the involvement of the GP in the transfer decision is low. This occurs also during working hours when the availability of physicians can be assumed. The lack of medical consultation and differences in the organizational procedures of NHs may therefore contribute to potentially avoidable hospital transfers. Further research is needed to explore nursing staffs’ reasons for this non-involvement and possible organizational procedures in detail. There is further a need for a close monitoring of health changes to improve care of NHRs. According to experiences of other countries, NH physicians or specialized nurses can have a positive impact on managing symptoms on-site which might reduce burden transfers to hospital. In more than half of the transfers, NHRs wishes for end-of-life care were unknown. However, relevant others as family members and legal guardians were not always actively involved in the transfer decision instead. Structured and regular ACP discussions might improve communication and can ensure that all nursing staff is aware of individual care wishes before an ambulance is called.

## Electronic supplementary material

Below is the link to the electronic supplementary material.

Supplementary file1 (Docx 17 kb)

## Data Availability

The datasets generated during and/or analyzed during the current study are available from the corresponding author on reasonable request.
