# Supplementary material for: Unplanned hospital transfers from nursing homes: who is involved in the transfer decision? Results from the HOMERN study
Source: Aging Clin Exp Res. 2020 Nov 30;33(8):2231–41. doi: 10.1007/s40520-020-01751-5 (PMC8302553; doi:10.1007/s40520-020-01751-5)
Supplement: Supplementary file 1 — Supplementary file1 (Docx 17 kb) [file 40520_2020_1751_MOESM1_ESM.docx]

**e-table 1: Reasons for unplanned hospital transfers**

|  | **Total** | | **Hospital admissions** | | **ED visits** | |
| --- | --- | --- | --- | --- | --- | --- |
|  | N=535 | (100%) | N=334* | (100%) | N=195* | (100%) |
| Deterioration of health status  (e.g. fever, infection, dyspnea, exsiccosis) | 188 | (35.1%) | 162 | (48.5%) | 22 | (11.3%) |
| Fall/accident/injury | 179 | (33.5%) | 76 | (22.8%) | 101 | (51.8%) |
| Psychiatric/neurologic disorders  (e.g. challenging behavior, stroke) | 38 | (7.1%) | 31 | (9.3%) | 7 | (3.6%) |
| Complications with catheter/tube  (e.g. blood in urine) | 38 | (7.1%) | 6 | (1.8%) | 32 | (16.4%) |
| Pain, not fall-induced | 33 | (6.2%) | 20 | (6.0%) | 13 | (6.7%) |
| Others  (e.g. gastrointestinal symptoms, bleedings) | 59 | (11.0%) | 39 | (11.7%) | 20 | (10.3%) |

*ED: emergency department*

**: Numbers differ due to missing values*
